# Assessment of the neuroprotective effect of *Cocos nucifera *L. oil on learning and behavior impairment in ovariectomized rats

**DOI:** 10.22038/AJP.2023.22724

**Published:** 2023

**Authors:** Ali Balderan, Yasamin Farrokhifar, Mahmoud Hosseini, Elnaz Khordad, Saeedeh Askarian, Samaneh Kakhki, Farimah Beheshti

**Affiliations:** 1 *Student Research Committee, Torbat Heydariyeh University of Medical Sciences, Torbat Heydariyeh, Iran*; 2 *Division of Neurocognitive Sciences, Psychiatry and Behavioral Sciences Research Center, Mashhad University of Medical Sciences, Mashhad, Iran*; 3 *Neurogenic Inflammation Research Center, Mashhad University of Medical Sciences, Mashhad, Iran*; 4 *Neuroscience Research Center, Torbat Heydariyeh University of Medical Sciences, Torbat Heydariyeh, Iran *; 5 *Departments of Physiology, School of Paramedical Sciences, Torbat Heydariyeh University of Medical Sciences, Torbat Heydariyeh, Iran *; 6 *Noncommunicable Diseases Research Center, Neyshabur University of Medical Sciences, Neyshabur, Iran*; 7 *Department of Clinical Biochemistry, School of Paramedical Sciences, Torbat Heydariyeh University of Medical Sciences, Torbat Heydariyeh, Iran*

**Keywords:** Cocos nucifera L., Morris water maze, Ovariectomy, Oxidative stress

## Abstract

**Objective:**

The current study aimed to investigate whether *Cocos nucifera* L. oil (CO) is effective on menopause-related memory dysfunction in ovariectomized (OVX) rats.

**Materials and Methods:**

Fifty healthy female Wistar rats were randomly selected and classified into five groups as control, OVX rats, and three OVX groups of rats which received three different doses (100, 200, and 400 mg/kg/day) of CO for five consecutive weeks by gavage. To assess the effect of CO, neurobehavioral tests such as Morris water maze (MWM) and Passive avoidance (PA) were done and then the animals were sacrificed to remove cortical and hippocampal tissues for biochemical analysis.

**Results:**

In both behavioral tests including MWM and PA, treatment with CO particularly two higher doses of 200, and 400 mg/kg demonstrated significant improvement in comparison with OVX group. Furthermore, antioxidant biomarkers such as total thiol content, catalase and superoxide dismutase (SOD) activities were significantly higher in the OVX-CO groups versus the OVX group. On the contrary, malondialdehyde (MDA) concentration as an oxidative stress biomarker was remarkably lower in the OVX-CO200 and 400 mg groups than the OVX group.

**Conclusion:**

The present study demonstrated the significant improvement of CO on learning and memory impairment induced by ovariectomy. Although the exact mechanism needs further investigation, it might have occurred due to the anti-oxidative effect of CO.

## Introduction

Menopause as a natural part of the aging process in women involves many organs and leads to a wide variety of complication including the increased risk of depression, osteoporosis, cardiovascular disease, severe cancers, and neurodegenerative diseases (Sherwin, 2005 ▶). The increasing concerns about various problems caused by menopausal phenomena are probably related to permanent cessation of the menstrual cycle and ovarian functions which leads to lack of estrogen and progesterone as important female hormones playing pivotal roles in the regulation of physiological process. Furthermore, there is strong evidence supporting that the shortage of estrogen due to menopause leads to reduction of endogenous antioxidant resulting in increased levels of oxidative stress mediators (Moreau et al., 2020 ▶). Besides, several studies demonstrated the negative effect of menopause on natural functions of the central nervous system (CNS) like learning-memory which is likely related to elevated level of oxidative stress and insufficient secretion of estrogen hormone (Asiaei et al., 2017 ▶; Delrobaei et al., 2019 ▶; Hogervorst and Bandelow, 2009 ▶). In fact, normal learning and memory process is attributed to appropriate level of estrogen (Hogervorst and Bandelow, 2009 ▶; Hojo et al., 2004 ▶) which was confirmed by recent studies on experimental menopause models (Kretz et al., 2004 ▶; Luine, 2014 ▶). In addition, there are three main estrogen receptors (Estrogen receptor alpha and beta (ERα and β), and G protein-coupled estrogen receptor (GPER)) in the hippocampus tissue (Kirshner et al., 2020 ▶; Vargas et al., 2016 ▶).

Also, many animal studies done either in rodents or non-human primate emphasized the significant effect of sex hormones especially estrogen on cognitive skills which was also confirmed by studies in ovariectomized (OVX) animals showing remarkable alteration in both structure and function of the hippocampus and cortical tissue accompanied by impaired animal performance in several cognitive tests (Ancelin and Ritchie, 2005 ▶; Ciappolino et al., 2018 ▶).

Regards evidences between reactive oxygen species (ROS) and cognitive disorder, so, in addition administration of antioxidant agents might be noteworthy to either treat or prevent many disturbances caused by menopause (Bargi et al., 2017 ▶; Kan et al., 2015 ▶; Pearson et al., 2015 ▶). *Cocos nucifera* L. oil (CO) which is extensively used in food and also recognized as a strong antioxidant could be an appropriate candidate to assess its therapeutic effect in learning and memory loss induced by menopause (DebMandal and Mandal, 2011 ▶; Feranil et al., 2011 ▶) . 

CO is predominantly consisted of different variety of triglycerides including short and medium-chain saturated fatty acids in addition to several biologically active polyphenol compounds which are not only strong inhibitors of lipid peroxidation, but also these fatty acids known as a strong neuroprotective agent especially in the prevention of β-amyloid neurotoxic effects (Ferreira et al., 2019 ▶; Laureles et al., 2002 ▶). Interestingly, there is around sixty percentage of similarity between the medium-chain triglycerides (MCT) in the CO with mother’s milk as an ideal natural nutrition for infants to protect from a wide variety of infections and diseases (Zou et al., 2016 ▶) and also lauric acid as a main component of MCT is widely administered for premature infants for improvement of brain development (Gardner et al., 2017 ▶). 

Thus, this study aimed to assess the effect of CO on OVX rats with learning and memory impairment. 

## Materials and Methods

**Animals and materials**

Fifty healthy female Wistar rats (8 weeks old, 180-200 g) were provided from the animal house of Torbat Heydariyeh University of Medical Sciences and placed into their cages equipped with standard condition (12/12 hours light/dark cycle and room temperature around 20-24^°^C) and they had free access to food and water. They were randomly divided into five groups (n=10/group) including control, ovariectomized rats (OVX), and OVX rats which received different doses of CO (100, 200, and 400 mg/kg) for five consecutive weeks via gavage.

All experimental protocols in the present study were first approved by the Research Ethics Committee of Torbat Heydariyeh University of Medical Sciences (Approval NO: IR.THUMS.REC.1399.020).

**OVX Procedure**

For ovariectomy, the animals were anesthetized using ketamine (90 mg/kg) and xylazine (10 mg/kg). For ventral incision, a decreased respiratory rate, and lack of response of foot pad to gentle pinch was necessary. Ventral incision in the middle of the abdomen was made through the muscle. Ovaries were clamped and removed. The abdominal walls were sutured and then animals were returned to their cages. The treatment was begun a week after the surgery (Hosseini et al., 2009 ▶). 

**Behavioral tests**

**Morris Water Maze (MWM)**

MWM as one of the most popular rodent model for assessing the spatial memory and learning was carried out. The apparatus includes a deep pool divided hypothetically into four quarters filled with water (23-25°C) and equipped with a hidden platform. The animals were allowed to swim freely four times from different position of pool for five consecutive days. A camera recorded all the movement, travel path, and distance and data was processed with software to report distance, and delay time to reach the hidden platform. Sixty seconds was given to each animal to search and find the platform and in the event that the animals were not successful to achieve the platform within the specific time period, they were gently directed. All the animals participated in the test were allowed to stay on the platform for 15 sec.

On day 6, probe test was performed. So, each rat was allowed to swim and find the platform’s location during 60 sec. The time spent in the target quadrant and path length were recorded (Vafaee et al., 2014 ▶).

**PA test**

The Passive Avoidance (PA) test is known as a standard animal model for assessing learning and memory. It includes two illuminated and dark place divided by a guillotine door. To explore the environment, the animals were allowed to move freely between the two spaces for five minutes and suddenly the guillotine door was closed and an electric shock (2 mA, 2 sec) was delivered to the foot of the rats as soon as they entered the dark room. According to experience acquired in the acquisition experiment, after 3, 24 and 48 hr, the animals were first placed in the light chamber, and both the delay in entrance and time stay in the dark part were recorded (Pourganji et al., 2014 ▶). 

**Biochemical assessment**

To assess the biochemical results, the day after the behavioral study, urethane as a common anesthetic agent was used to sacrifice the animals. For subsequent analyses of serum estradiol levels, blood samples were collected. The hippocampal and cortical tissues were removed as quickly as possible to analyze the total thiol content, malondialdehyde (MDA), superoxide dismutase (SOD), and catalase (Plastina et al.).

**Estradiol levels**

Elisa kit (MyBioSource) under the protocols of kit manufacturer was used to measure estradiol, so the blood samples were centrifuged at 3000 rpm for 15 min and sera were separated.

**MDA/total thiol concentration measurement**

MDA concentration as a well-known biomarker of lipid peroxidation along with total thiol content as one of the antioxidant indicator in both hippocampal and cortical tissues were measured according to the protocol described previously (Ghasemi et al., 2017 ▶; Khazdair et al., 2018 ▶). 

**Enzymatic analysis**

According to a previous report, the SOD and CAT activity was measured (Azizi-Malekabadi et al., 2018 ▶; Beheshti et al., 2019 ▶). Based on colorimetric techniques, the SOD activity was measured at 570 nm (Madesh and Balasubramanian, 1998 ▶). CAT activity was also measured (Aebi et al., 1976 ▶; Ahmadabady et al., 2021 ▶; Hosseini et al., 2018 ▶)

**Statistical analysis**

All the data are presented as mean±standard error of the mean. For statistical analysis, all data was first checked for normality and then biochemical data were analyzed by One-way ANOVA and Tukey's post hoc tests, while Two-way ANOVA test was used for behavioral tests. A p value<0.05 was considered statistically significant.

## Results

**Serum estradiol levels**

**Figure 1:**
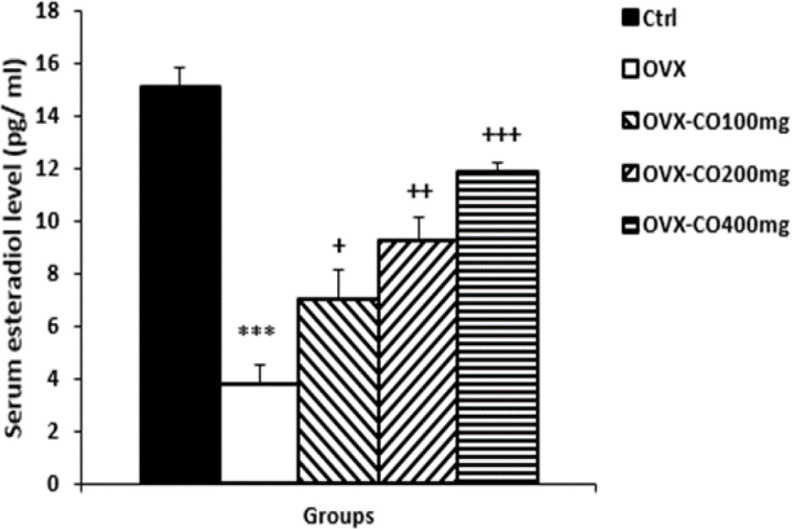
Serum estradiol level. Data are presented as mean±SEM, n=10. ***p<0.01 vs. the control group, +p<0.05, ++p<0.01 and ++p<0.01 vs. the OVX group

The OVX group demonstrated a significant lower level of serum estradiol compared to the control (p<0. 01), while treatment by CO at 100, 200 and 400 mg/kg increased this parameter (p<0.05- p<0.01- p<0.001, respectively; Figure 1).

**MWM**

**Figure 2:**
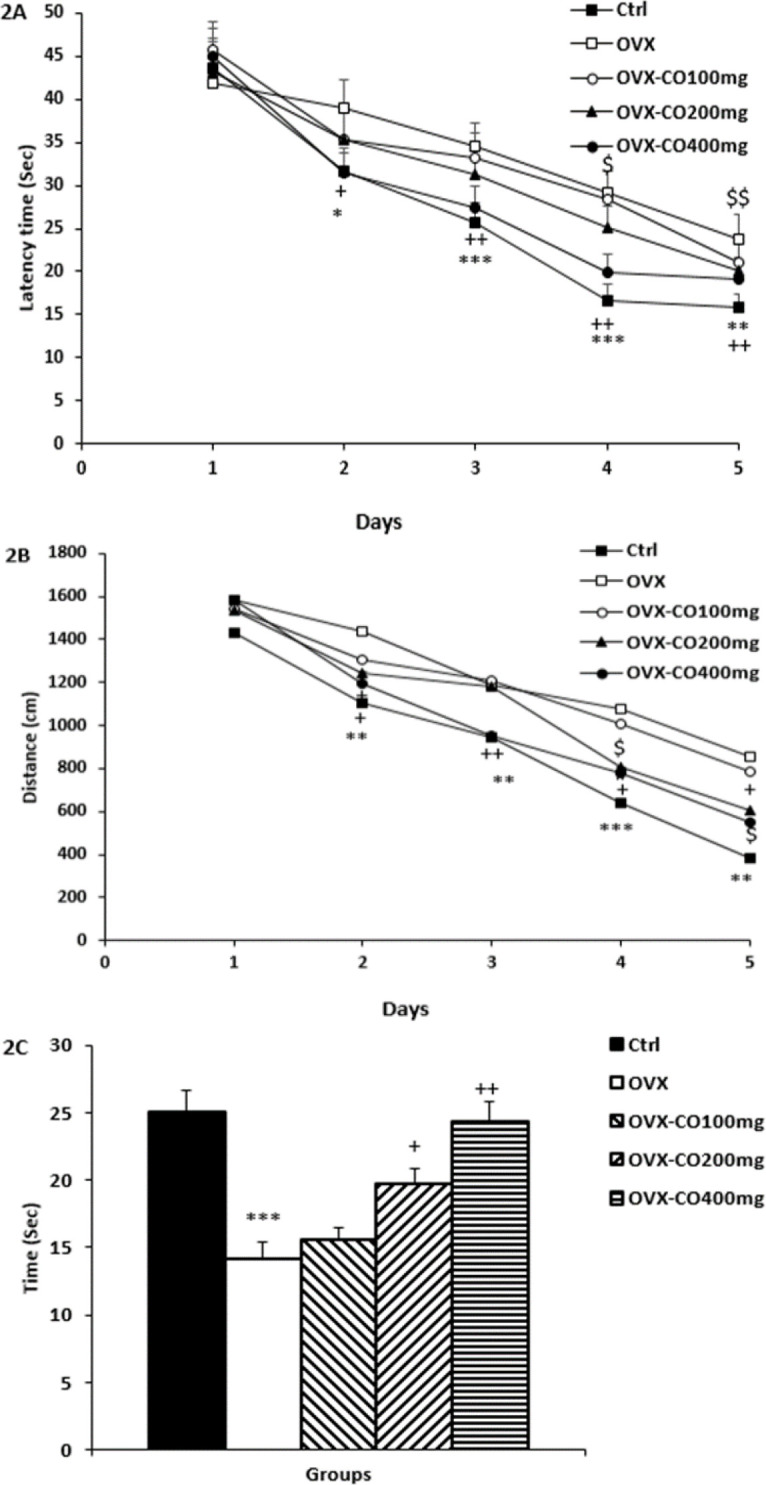
Comparison of latency (A), traveled distance (B) and time spent in the target quadrant (C). Data are presented as mean± SEM, n=10. *p<0.05, **p<0.01 and ***p<0.001 vs. the control group, $p<0.05, $$p<0.01, +p<0.05 and ++p<0.01 vs. the OVX group

According to statistical analysis, the time spent and path length traveled to reach the hidden platform in the OVX group were significantly higher than the control group day 2-5 (p<0.05, p<0.001, p<0.001 and p<0.01; Figure 2A, respectively for time spent and p<0.01, p<0.01, p<0.001 and p<0.01; Figure 2B, respectively for traveled distance).

Treatment by 200 mg/kg of CO decreased time spent and traveled distance to find the platform day 4 and 5 (p<0.05 and p<0.01; Figure 2A, respectively for time spent and p<0.05 and p<0.05; Figure 2B, respectively for traveled distance). Also, treatment by 400 mg/kg of CO decreased time spent and traveled distance to find the platform day 2- 5 (p<0.05, p<0.01, p<0.01 and p<0.01; Figure 2A, respectively for time spent and p<0.05, p<0.01, p<0.05 and p<0.05; Figure 2B, respectively for traveled distance).

On the probe day, the results showed that animals in the OVX group spent less time in the target quadrant than the control group (p<0.001; Figure 2C). However, CO 200 and 400 mg/kg, increased time spent in the target quadrant compared to the OVX group (p<0.05 and p<0.01; Figure 2C, respectively). 

**Passive avoidance test (PA)**

**Figure 3:**
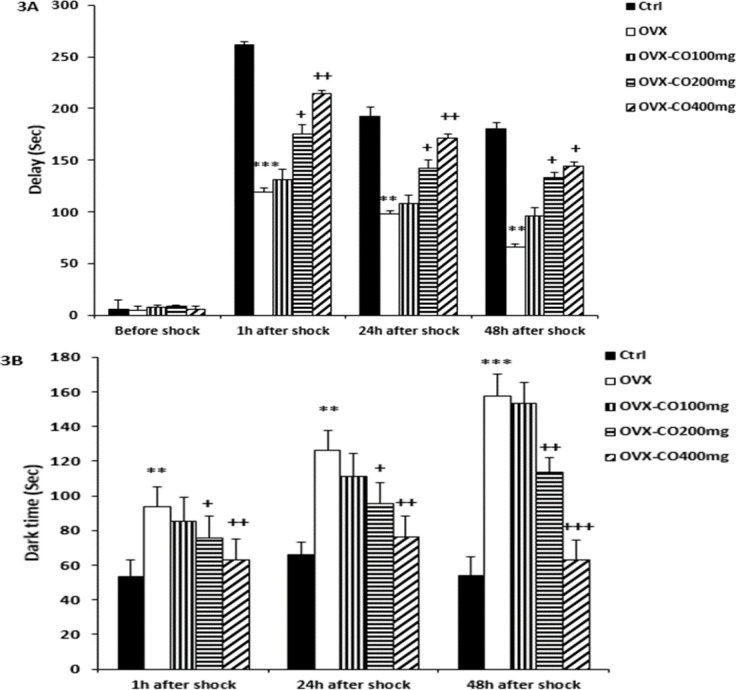
Comparison of latency (A) and the time spent in the dark compartment after the shock (B). Data are presented as mean±SEM, n=10. **p<0.01 and ***p<0.001vs. the control group, +p<0.05, ++p<0.01 and ++p<0.01 vs. the OVX group

The analysis of PA test data of the OVX group demonstrated significant difference with the control group in delay time to enter the dark chamber 1, 24, and 48 h after the shock (p<0.001, p<0.01 and p<0.01; Figure 3A, respectively). 

In 1, 24 and 48 h after shock, OVX-CO 200 (p<0.05 in all hours) and 400 mg/kg (p<0.05, p<0.05 and p<0.01; Figure 3A, respectively) groups showed significantly increased delay time to enter the dark chamber in comparison with the OVX group.

Also, the OVX group increased the time to stay in dark place compared to the control group 1, 24 and 48 h post shock (p<0.01, p<0.01 and p<0.001; figure 3B, respectively), while in 1, 24 and 48 h after shock, OVX-CO 200 (p<0.05, p<0.05 and p<0.01; Figure 3B, respectively) and 400 mg/kg (p<0.01, p<0.01 and p<0.001; Figure 3B, respectively) groups showed significantly increased time spent in the dark chamber in comparison with the OVX group. 

**Biochemical results**

**MDA concentrations and Thiol content in the hippocampal tissues**

**Figure 4:**
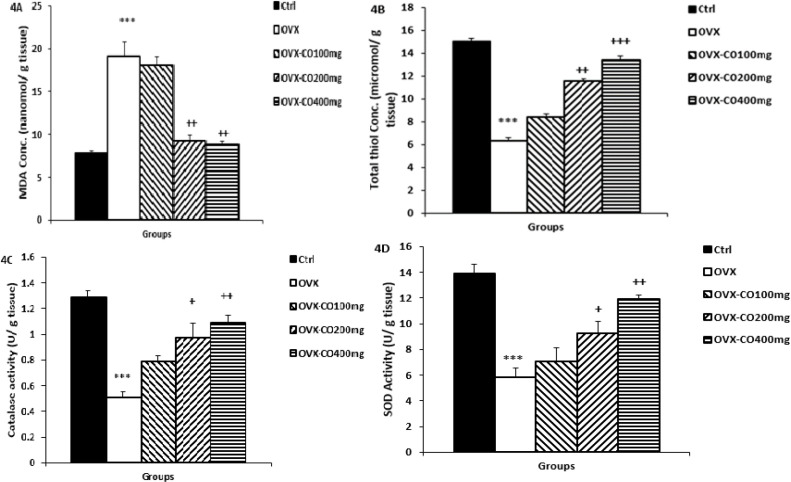
Hippocampal concentrations of MDA (A) and thiol groups (B) and hippocampal SOD (C) and catalase (D) activities. Data are presented as mean±SEM, n=10. *p<0.05 and **p<0.001 vs. the control group, +p<0.05, ++p<0.01 and +++p<0.001 vs. the OVX group

According to statistical analysis, MDA concentration was increased in the OVX group animals (p<0.001; Figure 4A), while total thiol content in the hippocampal tissue decreased compared to the control group (p<0.001; Figure 4B). Interestingly, pretreatment with CO at doses of 200, and 400 mg/kg attenuated the hippocampal MDA concentration (p<0.01 for both; Figure 4A), while augmented the hippocampal thiol content versus the OVX group (p<0.01, p<0.001; Figure 4B, respectively). 

**CAT and SOD in the hippocampal tissues**

The results of the current study showed that OVX led to a significant reduction in hippocampal CAT and SOD activity versus the control group (p<0.001 for both; Figure 4C and D), while the administration of two higher doses of CO including 200 and 400 mg/kg increased CAT activity (p<0.05, p<0.01; Figures 4C, respectively). Also, 200 and 400 mg/kg of CO increased SOD activity compared to OVX group (p<0.05, p<0.01; Figures 4D, respectively). 

**MDA concentrations and thiol content in the cortical tissues**

**Figure 5:**
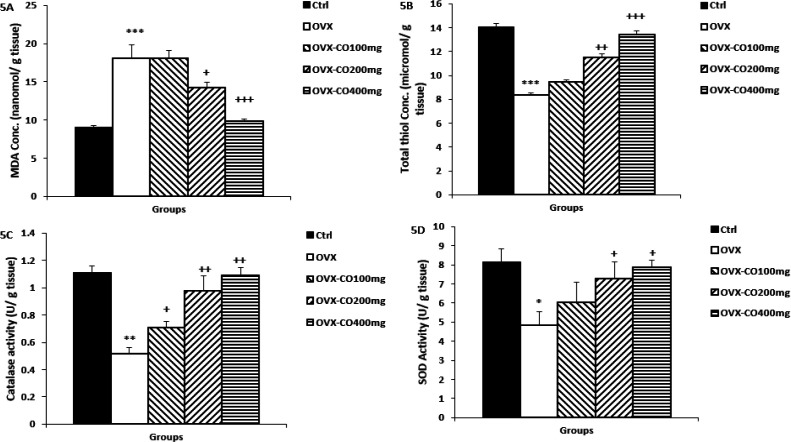
Cortical concentrations of MDA (A) and thiol groups (B) and cortical SOD (C) and catalase (D) activities. Data are presented as mean±SEM, n=10. *p<0.05, **p<0.001 and ***p<0.001 vs. the control group, +p<0.05, ++p<0.01 and +++p<0.001 vs. OVX group

Similar to hippocampal tissue, MDA and thiol had higher and lower concentration respectively, in the OVX group than the control group (p<0.001 for both; Figure 5A, Figure 5B), while CO 200, and 400 mg/kg significantly decreased cortical MDA compared to the OVX group (p<0.05 and p<0.001; Figure 5A, respectively). In addition, CO (200, and 400 mg/kg) increased cortical thiol compared to OVX group (p<0.01 and p<0.001; Figure 5B, respectively).

**CAT and SOD in the cortical tissues**

CAT and SOD activity was significantly decreased in cortex tissue of the OVX group compared to the control group (p<0.01and p<0.05, respectively), while CO extracts at all doses increased CAT activity compared to the OVX group (p<0.05 and p<0.01 for 200, and 400 mg/kg, respectively). 

## Discussion

The present hypothesis based on the positive antioxidant effects of CO especially at doses of 200, and 400 mg/kg on harmful impact of ovariectomy caused learning and memory impairment. The present study demonstrated the significant negative effect of estradiol deficiency on spatial learning and memory impairment by using behavioral tests including MWM and PA which induced by ovariectomy (Savonenko and Markowska, 2003 ▶). The spatial and non-spatial memory testes demonstrated significant decreases in rats 5 weeks after ovariectomy. So, it could be recommended that OVX rats in the current study could be used as a standard for memory loss in menopause. Additionally, ovariectomy models are commonly used for post-menopausal pathophysiological changes studies including cardiovascular disease, neurobehavioral disorders, osteoporosis, and so on. Several studies confirmed the strong relationship between learning and memory impairment caused by ovariectomy and elevated level of brain oxidative stress (Monteiro et al., 2005 ▶) which is in consistent with the present results.

The present study showed that in the OVX group, MDA was higher, while thiol concentration and SOD and CAT activities as antioxidant indicators were lower than the control group. In addition to lower level of serum estrogen, thus, these findings demonstrated that learning and cognition impairments observed in OVX rats might be due to lack of estrogen and deregulation of oxidative stress over antioxidant mediators leads to brain tissues damage. Therefore, amnesia caused by menopause can also be related to oxidative brain damage. Also, the results of other studies confirmed the strong relationship between that ovariectomy of animals or postmenopausal females with increased level of brain lipid peroxidation (Monteiro et al., 2005 ▶).

Antioxidant therapy in addition to hormone replacement therapy probably significantly prevent undesirable effect of menopause on many organs particularly the brain. Increasing research in recent years support the importance of diet and nutrition in reducing the symptoms of neurodegenerative disease like Alzheimer disease (AD) (Włodarek, 2019 ▶). CO included a wide variety of necessary triglyceride especially MCT and polyphenols providing beneficial effects in neurodegenerative disease like AD through a broad spectrum of pharmacological effects such as decreasing either oxidative stress mediators or pro-inflammatory cytokines (Vedin et al., 2012 ▶), which is consistent with present findings showed that CO improved learning and cognition deficiency of OVX rats in MWM and PA tests. To the best of author knowledge till now, there are no similar study demonstrating the effects of CO on learning and memory. Although, a similar study showed decrease the amyloid beta and increase the estradiol level in OVX mice using young coconut juice (Radenahmad et al., 2011 ▶). Another study demonstrated that CO improved learning and memory in colchicine-induced cognitive dysfunction model by antioxidative effects (John et al., 2020 ▶).

In the present study, the low level of estradiol was found in the OVX rats and CO augmented the level of both estradiol and antioxidant indicators, which is consistent with a previous study (Talboom et al., 2008 ▶). Furthermore, coconut water increased serum estrogen level in ovariectomized mice (Chomchalow, 2013 ▶). Therefore, considering scarce evidence on pathological effect of CO, and its natural compounds, using the CO in post-menopausal female is highly recommended to not only increase serum estrogen and antioxidant agents, but also prevent the brain damage and other associated disorders. 

According to the strong evidence supporting the beneficial effect of CO on brain and negative impact of menopause on spatial learning and memory, adjuvant therapy with CO to prevent some related disorder especially neurodegenerative disease caused by menopause, is highly recommended. 

## Conflicts of interest

The authors have declared that there is no conflict of interest.
